# An Overview of Different Interdental Cleaning Aids and Their Effectiveness

**DOI:** 10.3390/dj7020056

**Published:** 2019-06-01

**Authors:** Ethan Ng, Lum Peng Lim

**Affiliations:** 1 Discipline of Periodontics, Faculty of Dentistry, National University of Singapore, 11 Lower Kent Ridge Road, Singapore 119083, Singapore; denlimlp@nus.edu.sg; 2 Department of Restorative Dentistry, National Dental Centre Singapore, 5 Second Hospital Avenue, Singapore 168938, Singapore

**Keywords:** dental devices, interdental, oral health, dental plaque

## Abstract

Optimisation of plaque control is essential for the success of non-surgical and surgical periodontal therapy. This cannot be achieved with brushing alone; hence, there is a need for adjunctive interdental cleaning aids. The aim of this paper is to provide an overview of different interdental cleaning aids and review the literature for consensus on their effectiveness. A literature search of articles in English, up to December 2018, was conducted in Pubmed. High-quality flossing is difficult to achieve, and ineffective routine use of floss may not confer significant benefits over brushing alone. Interdental brushes are more effective than brushing as a monotherapy. They are at least as good if not superior to floss in reducing plaque and gingivitis. Although they are effective for patients regardless of their periodontal status (healthy or active), they are especially indicated in periodontal patients where widened embrasures are common. Added benefits include ease of use, patient acceptance, and recontouring of interdental tissues. Rubberpiks do not demonstrate inferiority to conventional interdental brushes. Wooden interdental aids appear to offer no significant advantage over brushing with respect to plaque removal; they may, however, reduce gingival bleeding. Oral irrigators are a promising tool for reducing gingival inflammation, despite minimal changes to plaque levels. For cleaning around dental implants, oral irrigators and interdental brushes are preferred over floss.

## 1. Introduction

A patient’s ability to achieve good mechanical plaque control is vitally important. Scaling and root planing without effective plaque control during the healing/maintenance phase results in subgingival recolonisation within 4–8 weeks [1]. Conversely, good supragingival plaque control appears to be sufficient in preventing relapse or recurrence of the disease due to subgingival recolonization [2]. Poor oral hygiene is also a significant risk factor for unsuccessful periodontal surgery [3], stability of guided tissue regeneration results [4], and peri-implant disease [5]. Today, although toothbrushing is the most common method of mechanical plaque removal, we may still not be very good at it. A systematic review by van der Weijden et al. found that, in adults with gingivitis, self-performed mechanical plaque removal with a manual toothbrush was not sufficiently effective [6]. More frequent tooth cleaning (up to twice daily) was shown to significantly improve gingival heath [7]. The reality is that brushing alone may only remove up to 60% of overall plaque at each episode of cleaning [8]. A more recent systematic review by Slot et al. estimated that the efficacy of plaque removal following a brushing exercise averages around 42% [9]. Brushing is also thought to be more optimal for cleaning facial surfaces of teeth compared to interproximal surfaces [10]. This is significant because interdental sites present the highest risk of plaque accumulation, whether anteriorly or posteriorly in the mouth [11]. Thus, interproximal surfaces of molars and premolars, being the predominant sites of residual plaque, are at higher risk of developing periodontal lesions and caries [12,13]. Clinically, gingivitis and periodontitis are usually more pronounced in interproximal areas than facial aspects [13].

Carefully performed supragingival plaque control was shown to be capable of altering the quantity and composition of both supragingival and subgingival microbiota. This was demonstrated by Dahlen et al., who found that, 24 months after initiation of a supervised oral hygiene program, total viable counts of bacteria in deep and shallow pockets and important bacteria species such as *Porphyromonas gingivalis* and *Aggregatibacter actinomycetemcomitans* decreased in number [14]. To aid in plaque control, various interdental cleaning aids are used. These include dental floss, interdental brushes, wooden interdental aids, and oral irrigators. A recent study by Marchesan et al. provides convincing data to support the use of interdental cleaning devices for promoting good oral health outcomes. The study found that interdental cleaning is associated with less periodontal disease, less coronal and interproximal caries, and fewer missing teeth; a higher frequency of interdental cleaning 4–7 times per week) was also associated with less interproximal periodontal disease [15]. These findings are in agreement with Crocombe et al., who found that regular interdental cleaning was associated with less plaque, calculus, and gingivitis [16]. The aim of this paper is to provide an overview of different interdental cleaning aids and review the literature for consensus on their effectiveness

## 2. Materials and Methods

A literature search was conducted in Pubmed using the Pubmed clinical query tool in December 2018. The keywords (interdental) AND (cleaning) were used, together with filters on category (therapy) and scope (broad). The search yielded 105 articles published between 1974 and 2018. Related articles in English, including reviews, meta-analyses, and clinical trials in humans, were included. Studies utilising interventions other than interdental cleaning aids (e.g., mouth rinses or subgingival irrigation), new products not commonly available in the market, and orthodontic subject populations were excluded. Abstracts were screened for relevance and full text was assessed for relevant articles. A manual search of the reference section of retrieved articles was also performed.

## 3. Dental Floss

Routine use of dental floss is low, ranging between 10% and 30% among adults [17]. The low compliance observed among adults could be because flossing is a technically challenging task. Studies showed that few individuals floss correctly and patients find flossing difficult, especially in areas with tight contact points [18,19,20]. Consequently, it was found that unsupervised flossing does not result in substantial reductions in gingival inflammation [21].

There is some evidence in the literature that the use of floss as an adjunct to brushing is potentially ineffective (Table 1). A review conducted by Berchier et al. concluded that routine instruction to use floss is not supported by evidence [22]. The results of a Cochrane review conducted in 2011 found “weak and very unreliable evidence” that flossing as an adjunct to brushing may be associated with a small reduction in plaque, although they did note a significant benefit with reducing gingivitis [23]. These findings are consistent with a meta-review in 2015, which states that most available studies fail to demonstrate the effectiveness of flossing in plaque removal, potentially due to technical difficulty or lack of patient compliance [24]. Indeed, adjunctive use of floss did not contribute further to plaque reduction compared to toothbrushing alone, even in young patients with intact papilla [25,26].

Despite substantial evidence citing a lack of support for the effectiveness of flossing in plaque removal, flossing may still confer benefits. Flossing itself is not harmful and has no major associated health risks, in addition to occasional short-term soft-tissue trauma [27]. Professional flossing was shown to be effective in reducing interproximal caries risk; however, this beneficial effect was lost when flossing was self-performed [28]. For patients lacking dexterity or compliance, floss holders represent a potential alternative. Studies demonstrated similar effectiveness of floss holders compared to handheld floss in reducing interproximal plaque and gingivitis [29,30]. They may also benefit patients lacking the dexterity to use hand floss [29]. Further, floss holders are significantly more effective in helping patients establish a long-term flossing habit, with floss holder users more likely to floss than hand-flossers [31]. It should be noted, however, that these studies comparing floss with floss holders did not include a control group for brushing. Reductions in interproximal plaque and gingivitis from pre-treatment levels are reasonable expectations, and this may not have differed when comparing the results to a group using brushing alone. Hence, most studies do not support the routine use of floss. Reductions in plaque or gingivitis can only be expected if patients can achieve high-quality flossing regularly. However, this may be an unrealistic expectation.

## 4. Interdental Brushes (IDBs)

### 4.1. Adjunctive Use of Interdental Brushes

Interdental brushes were investigated as early as 1976, where it was found that they were effective in removing plaque as far as 2–2.5 mm below the gingival margin [32]. They consist of a central metal wire core, with soft nylon filaments twisted around. The effectiveness of interdental brushes is well documented. One of the consensus findings from the European Federation of Periodontology 2015 workshop states that “cleaning with interdental brushes is the most effective method for interproximal plaque removal, consistently associated with more plaque removal than flossing or woodsticks” [33]. Two systematic reviews found that the adjunctive use of interdental brushes results in significant improvements on clinical parameters such as plaque scores, bleeding scores, and probing depth, when compared to brushing alone [34,35]. Another review by Salzer et al. found that interdental brushes were the most effective method for interdental plaque removal, compared to other interdental cleaning aids [24]. The superiority of interdental brushes is thought to be due to higher efficacy of plaque removal and high patient acceptance, as well as ease of use [10,26,33,36]. Thus, it is clear that the use of interdental brushes as an adjunct provides a clinical benefit over brushing alone.

### 4.2. Choosing an Appropriate Interdental Brush

There are several factors to consider when choosing an interdental brush which may affect its efficacy. The first is that size matters. In 2011, Imai et al. conducted a study which used a measuring tool to determine the best-fitting interdental brush for proximal sites. A colour-coded probe was inserted horizontally from the buccal aspect until snug, with the resulting colour on the probe corresponding to the matching colour of interdental brush. The reduced number of bleeding sites noted in this study was attributed to the use of an appropriate size of interdental brush, which resulted in “effective disruption of proximal biofilm compared to one size for all proximal spaces” [36]. Bourgeois et al. also used a colour-coded probe to determine the appropriate size of interdental brush. They found that calibrated interdental brushes reduce interdental bleeding by 46% at one week and 72% at three months [37]. These findings are consistent with the opinion of other authors, who observed that the size of the interdental brush should fit snugly in the interdental space [34]. Most studies neither discussed interdental brush sizes used, nor indicated if interproximal brushes were used at all available proximal sites [34]. Failure to use an appropriate size of interdental brush may account for the lack of statistical difference between various interdental cleaning aids in other studies [36].

Another factor to consider is the geometry of the interdental brush. Straight interdental brushes may be more effective in interproximal plaque removal than angled interdental brushes with a long handle. Findings from Jordan et al. 2014 showed that straight interdental brushes were statistically superior at plaque removal than angled brushes; they were also found to be significantly more effective for posterior teeth [38]. Waist-shaped interdental brush heads were also found to remove significantly more biofilm than straight interdental brushes, resulting in lower plaque scores [39]. This can be explained by the superior cleansing effect on buccal and lingual line angles of waist-shaped brushes [39]. A triangular or conical form of interdental brushes may also provide better adaptation to the interdental space [34].

The material of the interdental brush is also worthy of consideration. It was observed that the metal wire in the middle of interdental brushes can be uncomfortable for patients with sensitive root surfaces [40]. Rubber interdental brushes/picks are a more recent development and could be a viable alternative to conventional interdental brushes. They were shown to be as effective as regular interdental brushes (metal core), with further benefits of greater patient compliance and acceptance, in terms of comfort and willingness to buy the product [25,41,42].

Regarding the safety profile of interdental brushes, there is concern regarding their use at healthy sites with no attachment loss, potentially resulting in trauma [33]. Existing studies, however, did not find any associated gingival damage [38,43] or hard-tissue damage after the use of interdental brushes [43]. No attachment loss was found for patients using interdental brushes for over ten years [32]. It should be noted that the interdental papilla is pressed downward while achieving its subgingival cleaning action [44]. This may help in recontouring interdental tissues, although there may be associated recession.

### 4.3. Floss vs. Interdental Brushes

Many studies investigated the use of interdental brushes compared with floss using various clinical parameters (Table 2). In periodontally healthy subjects, the adjunctive use of interdental brushes or rubber interdental picks resulted in lower interdental plaque scores compared to brushing alone; the same was not true in the group that used floss as an adjunct [25]. Interdental brushes were also found to be superior in reducing interproximal bleeding in healthy patients with filled embrasures [36]. In a population of patients with mild to moderate chronic periodontitis, bleeding on probing and probing depth were reduced over a month of follow-up when interdental brushes, but not floss, were used [45]. This study found that interdental brushes and floss did not differ statistically for removal of supragingival or subgingival plaque. Christou et al. investigated the use of interdental brushes vs. floss in untreated patients with moderate to severe periodontitis. Although they found no difference between floss and interdental brushes for bleeding indices, interdental brushes were associated with more effective plaque removal and greater pocket reduction [10]. These findings are consistent with another study conducted using a population of untreated chronic periodontitis patients. Changes in plaque, papillae level, and probing depths were found to be significantly greater in the interdental brush group than the floss group [44]. The superiority of interdental brushes over floss is also apparent in patients undergoing periodontal maintenance. This was demonstrated by two studies, which showed that interdental brushes (IDBs), when used as an adjunct to toothbrushing, are more effective in proximal plaque removal than floss [26,46]. However, not all the literature is in unanimous agreement. In 2013, a Cochrane review concluded that, although there is some evidence that IDBs reduce gingivitis at one month compared to flossing, there was insufficient evidence to claim additional benefits of interdental brushes as an adjunct for reducing plaque [35]. Thus, it can be surmised from existing evidence that, regardless of the periodontal status of the patient, interdental brushes appear to be superior (whether based on gingival index, plaque index, reduction in bleeding or probing, reduction in probing depths, or a combination) to floss. The use of interdental brushes for the removal of proximal plaque seems to be especially indicated in periodontal patients or patients on a maintenance program [10,26,44,46]. This could be because the bristles of an interdental brush are able to fill embrasures better and engage exposed irregularities in root surfaces.

Another clinical situation where interdental brushes may be superior is cleaning around implants. Peri-implantitis was shown to be associated with inadequate plaque control and prosthetic constructions that did not allow accessibility to oral hygiene measures. Similar to some studies in natural teeth, interdental brushes demonstrated greater efficacy in removing proximal biofilm around implants [47]. The application of floss to exposed rough surfaces of an implant may also lead to fraying and, ultimately, the development of peri-implant conditions; hence, using interproximal brushes or toothpicks may be preferable [48]. With an increase in the number of implants placed and the occurrence of peri-mucositis and implantitis, effective oral hygiene to remove biofilm is likely to play an important role in future clinical practice.

## 5. Wooden Interdental Aids

Woodsticks are designed for mechanical removal of proximal plaque, achieved by friction against proximal tooth surfaces [40]. Comparatively fewer studies investigating woodsticks or toothpicks exist (Table 3). Similar to interdental brushes, woodsticks are able to remove plaque up to 2–3 mm subgingivally by depressing the papilla [49]. They fit best into interdental spaces with a triangular cross-section and should not be confused with toothpicks. Toothpicks are round, allowing only point contact with the tooth surface and, thus, are more suited for removing food debris after a meal [18]. One study, however, demonstrated similar efficacy of supragingival plaque removal to woodsticks [50].

A systematic review of seven studies observed that woodsticks do not have an additional benefit on proximal plaque reduction compared to toothbrushing alone, although a reduction in gingival bleeding scores was noted [51]. Four of these studies compared the efficacy of woodsticks to floss, with three finding no significant difference in plaque scores and one favouring the use of floss. This result is similar to another early study which reported a comparable proximal plaque removal efficacy between toothpicks and floss [52]. Woodsticks may specifically remove subgingival plaque that is not visible; hence, gingival inflammation parameters may improve while minimally affecting the plaque index [51]. Indeed, Finkelstein et al. observed that, by disrupting interdental plaque, interdental cleaning aids can result in significant reduction in gingival inflammation, although they may appear to have minimal effects on visible tooth surface plaque accumulation [53]. Like interdental brushes, the use of woodsticks may require a certain amount of interdental space to be present. They seem the most appropriate for open interdental spaces in regions that are not too posterior in the mouth, due to their specific angle of entry into the embrasure [54]. The advantages of toothpicks/woodsticks include ease of use and convenience. They may be more acceptable to older patients, especially those who routinely use toothpicks to remove food debris after eating.

## 6. Oral Irrigators

Oral irrigators were first introduced to the dental profession in 1962 [55]. In 2001, the American Academy of Periodontology stated that “supragingival irrigation with or without medicaments reduces gingival inflammation beyond what is normally achieved by toothbrushing alone” [56]. The mechanical mode of action of oral irrigators is through a combination of pulsation and pressure. This provides phases of compression and decompression of gingival tissue, removing supragingival plaque and flushing out subgingival bacteria and other debris [57]. Two zones of hydrokinetic activity are created—an impact zone where the solution contacts the gingival margin, and a flushing zone where the irrigant reaches subgingivally [55].

In addition to their ability to flush away loosely adherent plaque, remove bacteria cells, and interfere with plaque maturation [58], the use of oral irrigators was shown to reduce inflammation by reducing pro-inflammatory cytokines (IL-1β and PGE_2_) in the gingival crevicular fluid of patients with localised mild to moderate periodontitis and diabetics [59,60]. Indeed, by altering specific host-microbial interactions in the subgingival environment, pulsations from oral irrigators may reduce inflammation independent of plaque removal [61]. Cobb et al. observed that oral irrigators facilitate removal of subgingival biofilm up to 6 mm, inducing qualitative changes in subgingival plaque while not harming soft tissue [62]. The depth of penetration of oral irrigation was also measured in other studies. Indeed, 90% pocket penetration was achieved when probing depths were ≤6 mm [63]; oral irrigators were found to penetrate on average about half the depth of pockets, with the greatest penetration for pockets 0–3 mm and >7 mm [64]. The use of an oral irrigator does not appear to require any special motor skill, as demonstrated in 5.5–6.5-year-old children [65].

Attached gingiva can withstand pressure up to 160 psi for 30 s without irreversible damage, leading Bhaskar et al. to recommend that 90 psi is acceptable on undamaged tissue and 50–70 psi suitable for inflamed or ulcerated tissue [57]. A position paper by the American Academy of Periodontology supports the use of supragingival irrigation at forces of 80–90 psi, also commenting that oral irrigation poses no safety hazard as bacteraemia levels are similar to toothbrushing, flossing, scaling, and chewing [66]. Information concerning higher-risk patients who require prophylaxis prior to periodontal therapy is unavailable.

Several studies exist supporting the efficacy of oral irrigators as compared with other interdental devices (Table 4). Most of the studies were generated using commercially available products like Waterpik and Philips Sonicare Airfloss. Most of the studies demonstrated that oral irrigators may be more effective than dental floss or interdental brushes in reducing bleeding, plaque, or probing depths [59,60,67,68,69,70,71,72,73,74,75]. The utility of oral irrigators can also be extended to implant maintenance as shown by Magnuson et al., who observed that the water flosser group had significantly greater bleeding reduction than floss cleaning around implants [76].

A systematic review of seven included studies published between 1971 to 2000 showed that oral irrigation does not have a beneficial effect in reducing visible plaque, although there is a trend favouring improvement in gingival health compared to toothbrushing alone [77]. Indeed, some studies discussed showed improvement in plaque index parameters while others did not. Secondary subgingival penetration after supragingival irrigation may explain why gingival inflammation is frequently diminished, despite unchanged plaque levels [66]. Thus, the benefit of oral irrigators seems to be reducing parameters of gingivitis, and this effect may not be related to plaque removal.

## 7. Summary

Interdental cleaning aids play a vital role in optimising gingival health and preventing oral disease. Based on the results of this review, interdental brushes provide a significant benefit over brushing as a monotherapy. The use of floss may not achieve similar results if not effectively performed. Regarding gingival and plaque indices, interdental brushes may be superior to dental floss in at least one parameter, with the added benefits of patient comfort and acceptance. They are especially indicated in periodontal patients, who are likely to have widened embrasures. Rubberpiks were shown to be comparable to interdental brushes for reducing gingivitis and plaque. The biphasic mode of action of oral irrigators may result in qualitative changes in subgingival plaque. They may, thus, reduce gingival inflammation, although overall plaque levels as measured supragingivally may appear unaffected. Wooden interdental aids appear to offer no significant reduction in plaque compared to brushing, although they may reduce gingival inflammation. Interdental brushes and oral irrigators are recommended over floss for implant maintenance. We provided an overview of different interdental cleaning aids and their effectiveness. However, there is no single cleaning aid that works best for all patients. The option of an appropriate interdental cleaning aid is also influenced by the ease of use, size of interdental space, acceptability, dexterity, and motivation of the individual.

## Figures and Tables

**Table 1:** Summary of included studies on the effectiveness of flossing

Study	Study Design and Evaluation Period	Intervention	Primary Outcome Measures	Outcome
Berchier et al. [22]	Systematic review, 11 studies, 559 subjects, 1–6 months	Dental floss as an adjunct to brushing	Plaque index, gingival index	Flossing provides no additional benefit to brushing for the removal of plaque and reduction of gingivitis
Sambunjak et al. [23]	Systematic review, 12 studies, 1083 subjects, 1–6 months	Dental floss as an adjunct to brushing	Plaque index, gingival index	Significant benefit associated with flossing as an adjunct in reducing gingivitis. Insufficient evidence for reducing plaque
Kiger et al. [26]	Crossover study, 30 previously treated periodontal subjects, 3 months	Brushing vs. brushing + floss or interdental brushes as an adjunct	Gingival index, proximal plaque scores, buccal and lingual plaque index	The use of floss as an adjunct did not result in significant differences for any of the parameters
Graziani et al. [25]	Randomised clinical trial, 60 periodontally healthy subjects, 1 month	Brushing vs. brushing + floss or interdental brushes or rubber interdental brushes	Full-mouth bleeding scores, full-mouth plaque scores, probing pocket depth, recession, angulated bleeding score	The use of floss did not result in significant benefits over brushing alone
Blanck et al. [30]	Two-phase crossover study, 26 subjects, study duration not mentioned	Floss vs. floss holder	Plaque index	Both products removed statistically significant amounts of plaque compared to pre-treatment levels
Kleber et al. [31]	Survey, 32 respondents, 6 months after completion of a flossing study	Floss vs. floss holder	Number of participants flossing regularly and with which method after study completion	Floss holders are more effective in helping patients establish a regular flossing habit
Pucher et al. [29]	Comparative study, 36 dental students and 26 adult patients undergoing periodontal maintenance, 6 weeks	Floss vs. floss holder	Plaque index, gingival index	Both methods are effective in reducing interproximal plaque and gingivitis

**Table 2:** Summary of included studies on the effectiveness of interdental brushes and rubberpiks.

Study	Study Design and Evaluation Period	Intervention	Primary Outcome Measures	Conclusion
Imai et al. [36]	Randomised clinical trial, split mouth, 30 subjects, 12 weeks	Floss vs. IDB	Plaque index, bleeding index	No difference between both methods for plaque removal. IDBs are superior for reducing bleeding
Noorlin et al. [45]	Clinical trial, split mouth, 10 subjects with untreated periodontal disease, 1 month	Floss vs. IDB	Approximal plaque scores, bleeding on probing, probing depth	Both devices result in similar beneficial effects on plaque scores. Bleeding and mean probing depth reduction was significantly reduced compared to floss sites
Christou et al. [10]	Randomised clinical trial, split mouth, 26 subjects with untreated periodontal disease, 6 weeks	Floss vs. IDB	Plaque scores, probing depth, periodontal pocket bleeding index, angulated bleeding index	No difference between floss and IDB for bleeding indices. IDBs are more effective in plaque removal
Jackson et al. [44]	Randomised clinical trial, 77 subjects with untreated periodontal disease, 12 weeks	Floss vs. IDB	Plaque index, bleeding index, bleeding on probing at interdental sites, relative interdental papillae level	More improvements in the IDB group for every parameter
Kiger et al. [26]	Crossover study, 30 previously treated periodontal subjects, 3 months	Brushing only vs. brushing + floss or IDB as adjunct	Gingival index, proximal plaque scores, buccal and lingual plaque index	IDBs as an adjunct are superior to floss for proximal plaque removal
Rosing et al. [46]	Comparative study, 55 subjects on a maintenance program, no follow-up period	Floss vs. IDB	Plaque index	IDBs are superior to floss for removal of interdental plaque
Graziani et al. [25]	Randomised clinical trial, 60 periodontally healthy subjects, 1 month	Brushing vs. brushing + floss or interdental brushes or rubber interdental brushes	Full-mouth bleeding scores, full-mouth plaque scores, probing pocket depth, recession, angulated bleeding score	Adjunctive use of IDBs or rubber picks reduces more interdental plaque than brushing alone or flossing
Hennequin-Hoenderdos et al. [42]	Randomised clinical trial, split-mouth, 42 subjects, 1 month	Conventional IDBs vs. rubber bristles	Bleeding on marginal probing, plaque index, gingival abrasion score	Rubber bristles were more effective in reducing gingival inflammation. They were associated with less gingival abrasion and better received by participants
Bourgeois et al. [37]	Randomised clinical trial, 46 subjects, 3 months	IDBs vs. brushing	Frequency of interdental bleeding	IDBs resulted in significantly less interdental bleeding (plaque scores not evaluated)
Abouassi et al. [41]	Randomised clinical trial, crossover design, 39 subjects, 1 month	Conventional IDBs vs. rubber bristles	Plaque index, bleeding index, patient satisfaction questionnaire	Rubber bristles were as effective as conventional IDBs with the added benefit of more comfort
Jordan et al. [38]	Randomised clinical trial, 128 subjects, 12 days	Straight vs. angled IDBs	Modified proximal plaque index	Straight IDBs were better at removing interproximal plaque, especially for posterior teeth
Chongcharoen et al. [39]	Randomised clinical trial + crossover study, 8 subjects post-initial periodontal therapy, 3 weeks	Straight vs. waist-shaped IDBs	Plaque index	Waist-shaped IDB had lower plaque scores than straight IDBs
Slot et al. [34]	Systematic review, 9 studies, 510 subjects, 1–3 months	IDBs as an adjunct to brushing	Plaque index, gingival index, bleeding on probing, probing depth	IDBs as an adjunct to brushing showed significant reductions in plaque scores, bleeding scores, and probing depth
Poklepovic et al. [35]	Systematic review, 7 studies, 354 subjects, 1–3 months	IDBs and flossing as adjuncts to brushing	Gingival index, plaque index	Adjunctive use of IDBs results in significant improvements in gingival and plaque indexes compared to brushing alone. There is some evidence that IDBs reduce gingivitis at 1 month compared to flossing. More evidence needed for 3 months. No conclusions could be drawn for a difference for plaque index
Luz et al. [47]	Clinical trial, crossover study, 12 subjects, 2 months	Floss vs. IDB (around implants)	Plaque index	IDBs are more efficacious in removing proximal biofilm around implants

**Table 3:** Summary of included studies on the use of woodsticks and toothpicks.

Study	Study Design and Evaluation Period	Intervention	Primary Outcome Measures	Outcome
Hoenderdos et al. [51]	Systematic review, 7 studies, 438 subjects, 3–14 weeks	Woodsticks as an adjunct to brushing	Plaque index, gingival index	No significant advantage on plaque removal, but reduces gingival bleeding tendency
Schmid et al. [52]	Clinical trial, crossover study, 21 periodontally healthy subjects, 2 weeks	Toothbrush vs. floss vs. toothpicks	Plaque index	No differences for visible proximal surfaces. Brushing was superior on buccal surfaces. Toothpicks were as efficient as brushing on lingual surfaces
Zanatta et al. [50]	Clinical trial, split mouth, 15 subjects	Toothpicks vs. woodsticks	Plaque index	No differences found between round toothpicks and triangular woodsticks for supragingival plaque removal

**Table 4:** Summary of included studies on oral irrigators.

Study	Study Design and Evaluation Period	Intervention	Primary Outcome Measures	Outcomes
Cutler et al. [59]	Clinical trial, crossover study, 52 subjects, 2 weeks	Oral irrigator vs. brushing	Probing pocket depth, bleeding on probing, gingival index, plaque index, IL-1β and PGE_2_ levels	Improvement in all clinical parameters with oral irrigation. A reduction in pro-inflammatory cytokine profile in gingival crevicular fluid
Al-Mubarak et al. [60]	Randomised clinical trial, 52 diabetic subjects with adult periodontitis, 12 weeks	Oral irrigator vs. brushing	Modified gingival index, probing pocket depth, plaque index, clinical attachment level, bleeding on probing, reactive oxygen species generation, cytokines (TNF-α, IL-1β, IL-10, PGE_2_), and HbA1c	Significant reduction in plaque index, gingival index, bleeding on probing, and reactive oxygen species generation compared to brushing. Significant reduction in IL-1β, and PGE_2_ from baseline within test group
Newman et al. [68]	Multi-centre randomised clinical trial, 155 subjects receiving supportive periodontal therapy, 6 months	Regular oral hygiene vs. oral irrigation with water vs. oral irrigation with water and zinc sulphate solution	Bacterial measurements, gingival index, bleeding on probing	Oral irrigation with water was superior to regular oral hygiene and additional irrigation with zinc sulphate for reducing gingival inflammation. Oral irrigation with water significantly reduced bleeding on probing compared to regular oral hygiene
Barnes et al. [69]	Randomised clinical trial, 105 subjects, 1 month	Manual toothbrush + floss vs. manual toothbrush + oral irrigator vs. sonic toothbrush + oral irrigator	Bleeding index, gingival index, plaque index	Irrigation groups were more effective in reducing bleeding index and gingival index. The manual toothbrush + floss group was less effective than the sonic toothbrush + irrigation for reducing plaque
Rosema et al. [75]	Randomised home-use experiment, 108 subjects, 1 month	Oral irrigator vs. floss	Bleeding index, plaque index	Oral irrigator is superior to floss in reducing gingival bleeding
Goyal et al. [70]	Randomised clinical trial, 70 subjects, single use	Oral irrigator vs. floss	Plaque index	Oral irrigator as an adjunct is superior to floss as in plaque removal
Husseini et al. [77]	Systematic review, 7 studies, 590 subjects, 2–6 months	Oral irrigators vs. brushing	Plaque index, bleeding index, gingival index, probing pocket depth	Oral irrigators do not have a beneficial effect in reducing visible plaque. However, there is a trend of improving gingival health by reducing bleeding
Goyal et al. [71]	Randomised clinical pilot study, 28 subjects, two weeks	Oral irrigator vs. IDBs	Plaque index, bleeding on probing	Oral irrigators are more effective than IDBs for reducing gingival bleeding. No difference for plaque index
Lyle et al. [73]	Randomised pilot study, 28 subjects, single-use	Oral irrigator vs. IDBs	Plaque index	Oral irrigators as adjuncts remove significantly more plaque than IDBs after a single use
Stauff et al. [67]	Randomised clinical trial, 60 subjects, 4 weeks	Phillips Airfloss (microdroplet device) vs. floss	Papilla bleeding index, modified proximal plaque index, amount of gingival crevicular fluid	Microdroplet device was more effective at reducing plaque, with the added benefit of comfort of use
Goyal et al. [72]	Randomised clinical trial, 69 subjects, 1 month	Waterpik vs. Sonicare Air Floss Pro	Bleeding on probing, modified gingival index, plaque index	Waterpik is significantly more effective than Sonicare in reducing bleeding and gingivitis
Sharma et al. [74]	Randomised clinical trial, 82 subjects, single-use	Waterpik vs. Sonicare Airfloss	Plaque index	Waterpik removes significantly more plaque from tooth surfaces than Sonicare
Magnuson et al. [76]	Randomised clinical trial, 40 implants, 1 month	Oral irrigator vs. floss (around implants)	Bleeding on probing	Oral irrigator demonstrated significantly greater reduction in bleeding than floss
